# Afatinib helped overcome subsequent resistance to osimertinib in a patient with NSCLC having leptomeningeal metastasis baring acquired EGFR L718Q mutation: a case report

**DOI:** 10.1186/s12885-019-5915-7

**Published:** 2019-07-17

**Authors:** Jing Liu, Bo Jin, Hang Su, Xiujuan Qu, Yunpeng Liu

**Affiliations:** 1 Department of Medical Oncology, The First Hospital of China Medical University, Shenyang, China; 2 Key Laboratory of Anticancer Drugs and Biotherapy of Liaoning Province, The First Hospital of China Medical University, Shenyang, China

**Keywords:** Afatinib, EGFR, L718Q, Leptomeningeal metastasis, NSCLC

## Abstract

**Background:**

The epidermal growth factor receptor (EGFR)-mutated advanced non-small-cell lung cancer has been successfully treated with tyrosine kinase inhibitors (TKIs). Acquired resistance becomes a tough issue when patients fail to respond to the third-generation TKI osimertinib. This study aimed to report a case baring acquired EGFR L858R/L718Q mutation in the central nervous system induced by osimertinib, which was successfully overcome using afatinib.

**Case presentation:**

A 65-year-old female patient was diagnosed with stage IV non-small-cell lung adenocarcinoma with synchronic brain metastasis in February 2015. Before and during treatment, 416 tumor-related genes were monitored dynamically by liquid biopsies using next-generation sequencing, and the treatment strategy was decided according to the gene status. At baseline, an EGFR L858R mutation in exon 21 was detected, so treatment with icotinib was started. After 8 months, she experienced disease progression with leptomeningeal metastasis and switched to osimertinib based on an acquired EGFR T790 M mutation. After 9 months, her disease progressed and an EGFR L718Q mutation was found in the cerebrospinal fluid. The patient was then challenged with afatinib, and her disease was under control for 4 months. In January 2017, the patient passed away, with an overall survival time of 23 months, 15 months after leptomeningeal metastasis.

**Conclusion:**

The acquired EGFR L718Q mutation in the cerebrospinal fluid resulted in subsequent resistance to osimertinib and could be partly overcome using afatinib, indicating a promising treatment option in the clinic.

## Introduction

The EGFR-mutated advanced non-small-cell lung cancer (NSCLC) has been successfully treated by sequentially administering several tyrosine kinase inhibitors (TKIs). Acquired resistance becomes a severe issue when patients fail to respond to the third-generation TKI osimertinib. EGFR L718Q mutation has been reported recently as a rare mechanism of osimertinib resistance, but no therapy is effective enough to conquer it [1]. This study aimed to present a case of advanced NSCLC with leptomeningeal metastasis baring primitive EGFR L858R and acquired L718Q mutations in the cerebrospinal fluid (CSF), which was successfully treated using afatinib.

## Case presentation

### Patient information

A 65-year-old woman came to the clinic of the First Hospital of China Medical University in January 2015 with main complaints of moderate headache and mild nausea. Her medical history included hypertension and coronary disease for 10 years, which were under control with oral medications. She was a non-smoker and a non-drinker, and denied any family history of cancer.

### Clinical findings

Physical examination revealed a palpable nodule in the lower right cervical zone, which was about 1.0 cm in diameter, painless, hard, and fixed with surrounding structures. No other significant clinical findings were detected.

### Diagnostic assessment

**Fig. 1:**
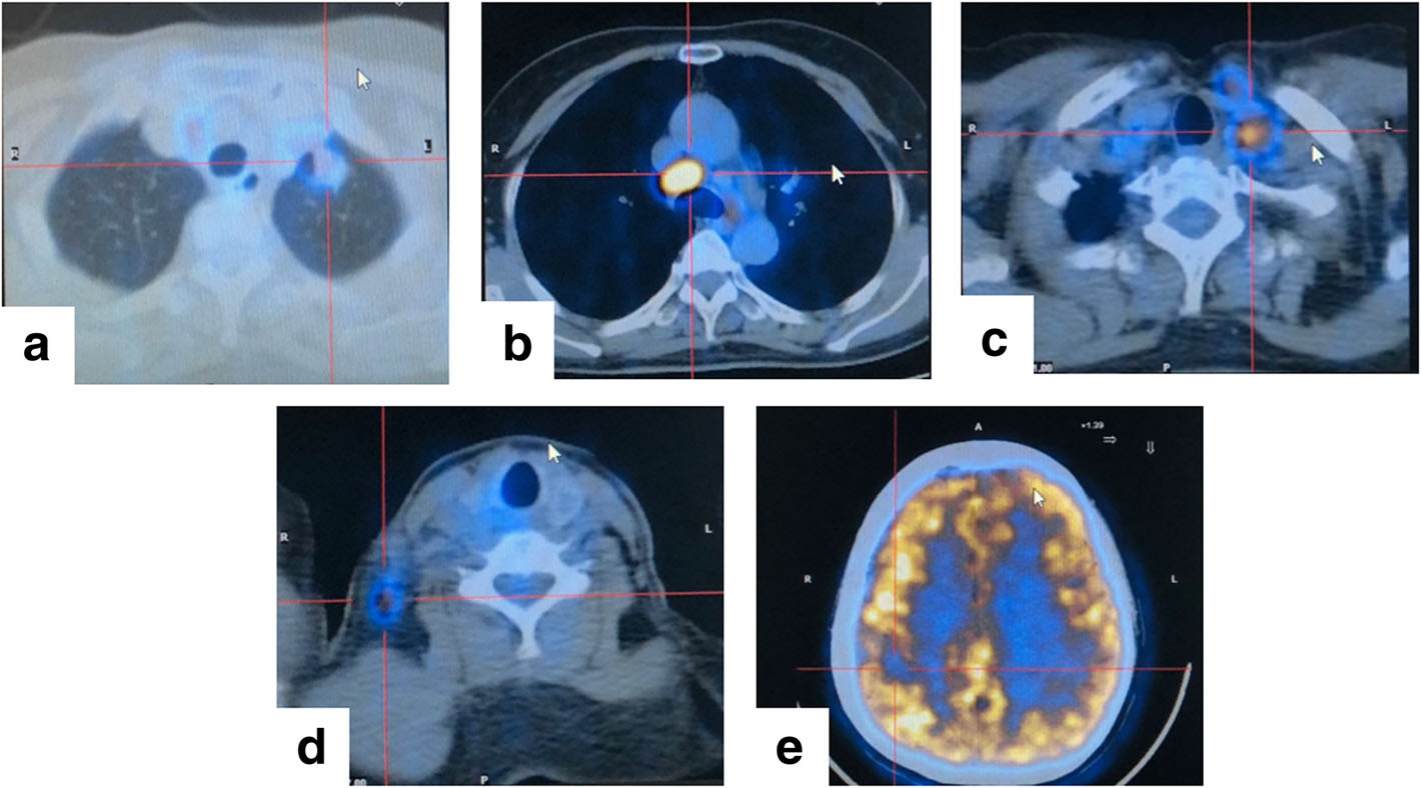
Positive finding by PET-CT scan at baseline. **a** Primary lung tumor, **b**–**d** metastatic lymph nodes, and (**e**) brain metastasis

Multiple enlarged lymph nodes in the right lower cervical and bilateral supraclavicular zone were detected by ultrasound. A chest computed tomography (CT) scan with contrast revealed a 1.5-cm nodule in the left upper lobe of the lung, with multiple enlarged lymph nodes in the left hilum and bilateral mediastinum. Brain magnetic resonance imaging showed a 1.2-cm nodule in the right parietal lobe with mild surrounded swelling. F^18^-Fluorodeoxyglucose (FDG) positron emission tomography CT (PET-CT) scan confirmed multiple lesions with increased FDG uptake (Fig. 1a–e).

An ultrasound-guided core needle biopsy of the cervical lymph node was performed in January 2015. Pathological and immunohistochemical (IHC) examinations indicated metastatic adenocarcinoma of the lung. The IHC staining revealed the following: carbohydrate antigen (CA)125 (+), CA199 (+), cluster of differentiation (CD) 34 (blood vessels+), caudal type homeobox 2 (CDX2) (−), cell keratin (CK) 19 (+), galectin-3 (±), Ki-67 (40%+), mammaglobin (±), naspin-A (+), paired box gene 8 (PAX8) (±), thyroglobulin (−), and thyroid transcription factor-1 (TTF-1) (+). EGFR gene detection showed L858R mutation in exon 21.

The patient was diagnosed with non-small-cell lung adenocarcinoma with clinical staging of T1N3M1b, stage IV (with synchronic brain metastasis), according to the American Joint Committee on Cancer version 7.0 [2].

### Therapeutic intervention

**Fig. 2:**
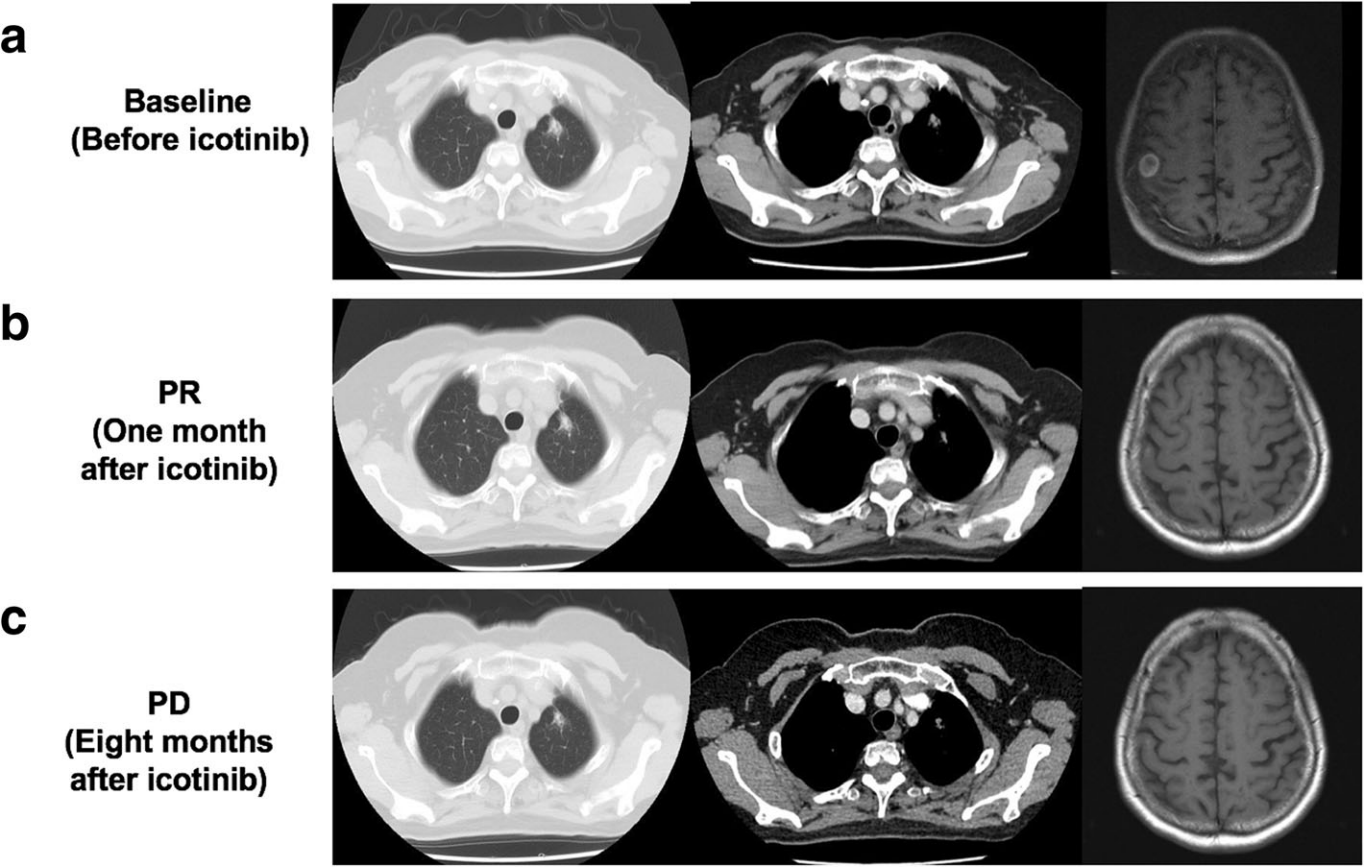
Clinical response to icotinib treatment. **a** Primary tumor (left and middle) and brain metastasis (right) at baseline before icotinib treatment. **b** Partial response after 1 month of treatment. **c** Progressive disease after 8 months of treatment with icotinib. PD, Progressive disease; PR, partial response

From February 2015, the patient started treatment with icotinib (125 mg tid orally). A partial response was achieved after 1 month (Fig. 2a and b). In October 2015, she complained of severe headache and nausea, and leptomeningeal metastasis was confirmed by CSF cytopathology. No progression of primary lung tumor or metastatic brain lesion was found at that time (Fig. 2c). Intrathecal chemotherapy with methotrexate was given (methotrexate 10 mg, every other day), and liquid biopsy using plasma and CSF was performed. Next-generation sequencing (NGS) reported EGFR gene amplification, primitive L858R mutation, and a new T790 M mutation in CSF, but not in plasma. Osimertinib was then administered (80 mg/day orally) from November 2015. The disease was controlled well, and all symptoms disappeared. Repeated NGS using CSF after 2 months of osimertinib treatment showed EGFR gene amplification and L858R mutation only, while T790 M mutation was undetectable.

**Fig. 3:**
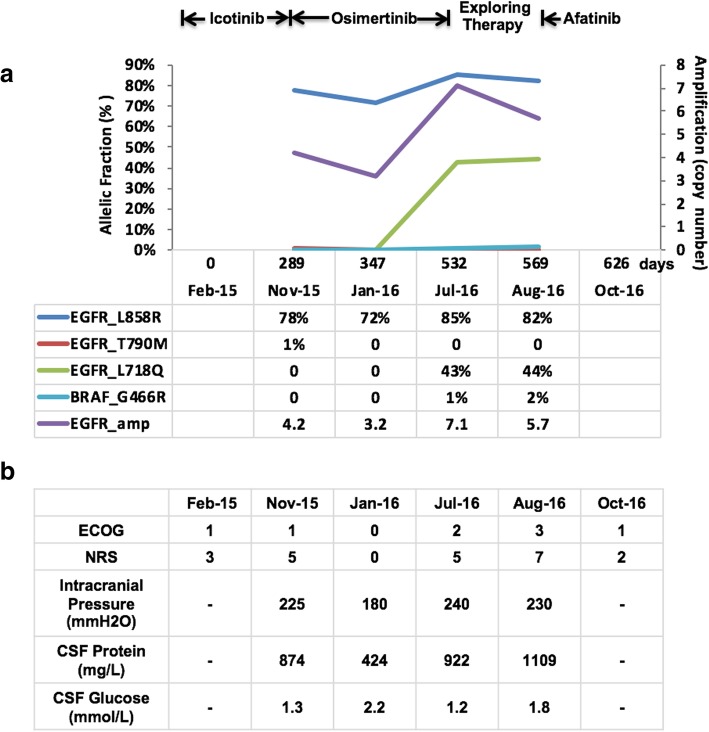
Dynamic changes in the allelic variations (**a**) and clinical factors (**b**). The *y*-axis represents the allelic fraction or amplification copy number, and the *x-*axis represents the treatment timeline. Each color represents a variant. CSF, Cerebrospinal fluid; ECOG, Eastern Cooperative Oncology Group; NRS, numeric rating scale

In July 2016, the patient developed disease progression and was admitted to the hospital in a critical condition. She presented with severe headache, dizziness, nausea, and vomiting, with persistent high intracranial pressure of 240 mm H_2_O. The Eastern Cooperative Oncology Group (ECOG) performance status was 3. Intense surveillance and life-supporting care were given. Liquid biopsy using CSF showed EGFR gene amplification, L858R mutation, and a new L718Q mutation. A b-rapidly accelerated fibrosarcoma (BRAF) G466R mutation was also found. Supportive care and exploring therapeutics were given sequentially after obtaining informed consent, including high-dose icotinib pulsatile therapy (375 mg every 3 days, followed by 625 mg every 5 days), temozolomide oral administration (200 mg/day for 5 days), and bevacizumab intrathecal injection (100 mg), as described in previous studies [3–6]. No treatment-related side effects were observed, and clinical evaluation showed no improvement in cancer-related symptoms, signs, or laboratory findings (Fig. 3a and b). The patient failed to respond to any of these treatments.

In August 2016, about 1 week after all the exploring therapeutics failed, afatinib was challenged (40 mg/day orally). The patient tolerated the treatment well with grade 1 diarrhea only. All the symptoms related to the disease improved gradually. After 1 month, she complained of only mild headache and nausea, and her ECOG performance status improved from 3 to 1. The dynamic changes in the allelic variations and clinical factors during treatments are shown in Fig. 3a and b.

### Follow-up and outcomes

**Fig. 4:**
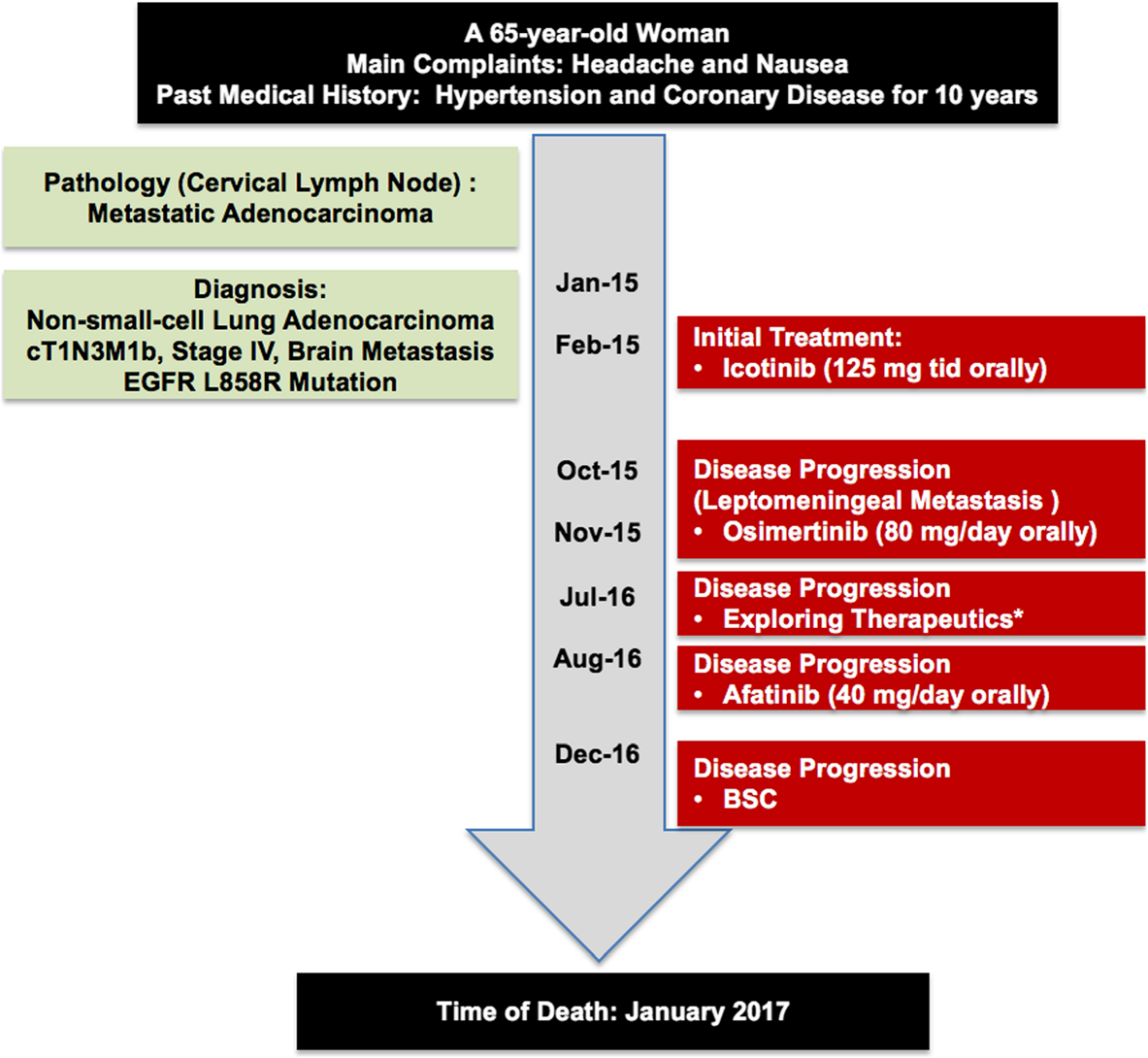
Treatment timeline. ^*^Exploring therapeutics included the following: high-dose icotinib pulsatile therapy, temozolomide oral administration, and bevacizumab intrathecal injection. BSC, Best supportive care

In December 2016, her symptoms became worse with disease progression. The patient passed away in January 2017. She survived for 23 months in total and for 15 months after suffering from leptomeningeal metastasis. The treatment timeline is shown in Fig. 4.

## Discussion and conclusions

Treatment of EGFR-mutated NSCLC with central nervous system (CNS) metastases using EGFR TKIs, which can penetrate the blood–brain–barrier, has achieved great success in recent years. Once a patient develops resistance to third-generation TKIs, the treatment options become very limited. Patients usually die soon after.

In the clinic, no experience after CNS failure to osimertinib has been reported. Chemotherapy combined with whole-brain radiation may be an option, but it is not suitable for most of the patients with leptomeningeal metastasis at poor performance status. Temozolomide has been reported showing a modest therapeutic effect as a single agent for refractory brain tumor or metastases, especially for patients unfit for intensive treatment [5]. Bevacizumab has also been reported to be effective in reducing malignant effusion and edema to relieve symptoms [6–8]. Based on these reports, the patient in the present study was challenged with temozolomide and bevacizumab, but unfortunately she did not respond to any of them.

The mechanisms of resistance to the third-generation EGFR TKIs have been demonstrated in recent years. The most common acquired mutations occur on codon 797 (i.e., C797S), while other rare mutations at position L718, L792, or G719 have also been reported recently [9, 10]. In vitro data showed that EGFR L718Q mutation conferred the highest resistance to osimertinib by affecting the conformation of the EGFR–osimertinib complex so as to prevent the reaction with C797 [11, 12]. An in vitro study showed that models harboring EGFR L858R/T790 M/L718Q were resistant to all EGFR inhibitors, but L858R/L718Q mutant remained sensitive to gefitinib and afatinib [13]. On the contrary, some other studies demonstrated that the EGFR L858R/L718Q variant was resistant to gefitinib in vitro [11].

High-dose icotinib and pulsatile erlotinib have been reported to exert better effects than the standard dose of first-generation TKIs in patients with NSCLC having refractory leptomeningeal metastasis [3, 4]. In the present case, the patient harbored EGFR L858R/L718Q and showed no response to high-dose pulsatile icotinib, but did respond to the standard dose of afatinib, indicating that the second-generation EGFR TKI had potential activity toward acquired EGFR L718Q mutation. Although only 4-month prolongation of survival was achieved by afatinib, the improvement in the quality of life was significant. Besides, for a patient with leptomeningeal metastasis refractory to all available therapeutics, a duration of 4 months was meaningful and promising.

In addition, a BRAF G466R mutation was detected while progressing upon osimertinib. BRAF V600E mutation has been reported to serve as the bypass mechanism for EGFR TKI resistance, but the contribution of non-V600E mutation is not clear [14]. No BRAF inhibitor was added to the treatment in the present case due to unknown effects of G466R mutation on downstream signaling.

This study presented clinical evidence on the efficacy of second-generation EGFR TKI targeting L858R/L718Q variant with CNS metastasis, indicating a potential treatment option in the clinic. The underlying mechanisms need to be further investigated.

## Data Availability

All data generated or analyzed during this study are included in this published article.

## Abbreviations

CNS: Central nerve system
CSF: Cerebrospinal fluid
CT: Computed tomography
ECOG: Eastern Cooperative Oncology Group
EGFR: Epidermal growth factor receptor
FDG: F^18^-Fluorodeoxyglucose
IHC: Immunohistochemistry
NGS: Next generation sequencing
NRS: Numeric rating scale
NSCLC: Non-small cell lung cancer
PET-CT: Positron emission tomography computed tomography
TKIs: Tyrosine kinase inhibitors
